# Molar loss further exacerbates 2-VO-induced cognitive impairment associated with the activation of p38MAPK/NFκB pathway

**DOI:** 10.3389/fnagi.2022.930016

**Published:** 2022-11-03

**Authors:** Yunping Lu, Qian Pang, Qianqian Wu, Bin Luo, Xiaofei Tang, Qingsong Jiang

**Affiliations:** ^1^Department of Prosthodontics, Beijing Stomatological Hospital, School of Stomatology, Capital Medical University, Beijing, China; ^2^Department of Stomatology, People’s Hospital of Beijing Daxing District, Capital Medical University, Beijing, China; ^3^Division of Oral Pathology, Beijing Institute of Dental Research, Beijing Stomatological Hospital, School of Stomatology, Capital Medical University, Beijing, China

**Keywords:** molar loss, vascular dementia, cognitive impairment, apoptosis, p38MAPK

## Abstract

**Background:**

Vascular dementia is characterized by reduced cognitive function due to chronic cerebral hypoperfusion and has become a significant public health challenge as the global population ages. Recent studies suggested that molar loss, a common problem among the elderly, may trigger the development of cognitive decline. Our previous study found that the molar loss affected cognitive dysfunction, and the astrocytes in the hippocampus of chronic cerebral ischemia rats were affected, but the underlying mechanism is unclear.

**Methods:**

In this study, we established the animal model of molar loss with 2-VO rats and the Morris water maze was used to test the cognitive ability of rats in each group. The damage to neurons was observed *via* Nissl staining, and neuronal apoptosis was analyzed by terminal deoxynucleotidyl transferase dUTP nick end labeling (TUNEL) assay in the hippocampus of the rats. Quantitative Real-Time PCR and immunohistochemistry and histology (IHC) were used to detect the expression of p38MAPK, NFκB, caspase 3, and iNOS in the hippocampus. The astrocytes were detected by IHC and Immunofluorescence analysis for GFAP. After 2-VO MO surgery, rats were administered DMSO or p38MAPK inhibitor (SB203580) by intrathecal injection.

**Results:**

The Morris water maze test showed that the molar loss aggravated spatial memory learning ability with chronic cerebral ischemia decreased in the rats. The neuronal damage and more apoptotic cells were observed in the hippocampus of 2-VO rats. After the molar loss, the mRNA and protein expression of iNOS, p38MAPK, NFκB, and caspase 3 were further upregulated in 2-VO rats. Molar loss upregulated GFAP expression, and the p38MAPK-positive cells were labeled with the astrocyte marker GFAP. SB203580 reduced cognitive impairment and apoptosis of hippocampal neurons in 2-VO rats following the molar loss.

**Conclusion:**

Molar loss can aggravate cognitive impairment in 2-VO rats to a certain extent. The mechanism of molar loss exacerbating the cognitive decline in 2-VO rats may be associated with the activation of the p38MAPK-NFκB-caspase 3 signaling pathway, which induces neuronal apoptosis.

## Graphical Abstract

Molar loss aggravates cognitive decline in 2-VO rats by the activation of p38MAPK-NFκB inducing neuronal apoptosis. 
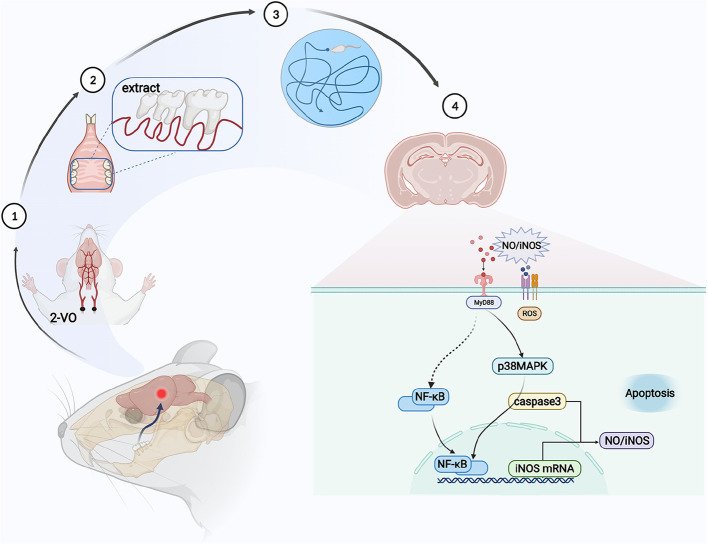

## Introduction

With the increasingly severe aging of society, tooth loss, a common problem among the elderly, has received attention progressively. The oral and maxillofacial system is responsible for physiological functions, such as chewing, swallowing, and speaking. An increasing number of relevant literature have manifested that molar loss affects cognitive function and is associated with dementia (Wang et al., 2022). The deterioration of their dental health was significantly increased in clinical dementia individuals (Okamoto et al., 2010; Cerutti-Kopplin et al., 2016; Orr et al., 2020). In animal models, loss of molars was found to reduce the number of pyramidal cells and acetylcholine levels in the hippocampus (Makiura et al., 2000; Kubo et al., 2005; Yamazaki et al., 2008). The study indicated that hippocampal structural remodeling can be induced by tooth loss in aged SAMP8 mice (Katano et al., 2020). Vascular dementia (VaD), the second most common form of dementia, is a progressive disease associated with age (Zhou T. et al., 2020). VaD is characterized by reduced cognitive function due to chronic cerebral hypoperfusion (CCH) and has become a major public health challenge as the global population ages (Hazalin et al., 2020). Vascular cognitive impairment (VCI) is a clinical stroke or evidence of subclinical vascular brain damage, and at least one cognitive area appears to be a syndrome of cognitive impairment (Gorelick et al., 2011). At present, the vascular risk factor is easy to detect and control, which is conducive to the early detection and treatment of cognitive impairment and dementia.

Chronic cerebral ischemia is one of the dementia risk factors. The hippocampal CA1 area is the functional area most closely related to learning and memory, and it is very sensitive to ischemia and hypoxia (Bird et al., 2018). Recent studies have shown that a rise in cerebral blood flow (CBF) was associated with mastication (Onozuka et al., 2003; Hasegawa et al., 2007). Tooth loss decreased CBF and increased glutamate, leading to cognitive impairment in rats (Luo et al., 2019). Our previous study found that the molar loss aggravated the existing cognitive dysfunction, and the astrocytes in the hippocampus of chronic cerebral ischemia rats were affected (Pang et al., 2015, 2020), but the underlying mechanism is unclear.

Activation of the p38 mitogen-activated protein kinase (p38MAPK) signaling pathway by CCH may initiate hippocampal neuronal apoptosis, leading to the cognitive dysfunction of VaD (Fang et al., 2005; Yang et al., 2013). MAPK is one of the central signal transduction pathways triggering neuronal apoptosis following neural hypoxia and reperfusion. p38MAPK affects apoptosis by regulating NFκB and caspase 3 expression (Ashabi et al., 2013). Ligation of bilateral common carotid arteries (2-vessel occlusion, 2-VO) is a classic model for investigating the mechanisms of VaD (Farkas et al., 2007; Du et al., 2017). Based on the 2-VO model, bilateral maxillary molars were extracted to simulate tooth loss. We investigated whether molar loss reduced cognitive function in 2-VO rats in the present study. Moreover, our results provide a possible underlying mechanism by which the molar loss aggravated VaD through p38MAPK/NFκB. Currently, the pathobiological mechanism of tooth loss on cognitive dysfunction is unclear. It is essential to clarify whether molar loss aggravates VCI and explore its influencing mechanism.

## Materials and methods

### Animal

**FIGURE 1:**
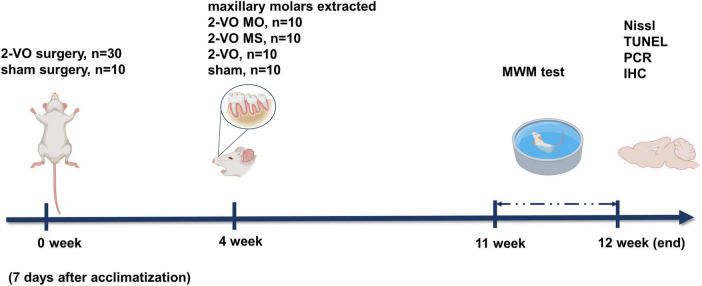
Schematic of the experimental protocol.

All animal experiments were carried out according to the Beijing Municipality on the Review of Welfare and Ethics of Laboratory Animals guidelines. The animal procedures were approved by the Animal Ethical and Welfare Committee of Beijing Stomatological Hospital (Approval No. KQYY-201509-001). Male Wistar rats (200 ± 50 g, 3 months old) were randomly divided into four groups (each group 10 rats) as follows: chronic cerebral ischemia sham operation group (sham group), chronic cerebral ischemia group (2-VO group), molar loss with chronic cerebral ischemia group (2-VO MO group), and molar loss sham with chronic cerebral ischemia (2-VO MS group). The experiment schedule is shown in Figure 1. At the same time, the molar loss group (MO group) and the control group (Ctr group) were also set up as controls. The results of the MO group and Ctr group were provided as Supplementary Figures 3–5.

### Surgery

Animals underwent two-vessel occlusion or sham surgery with 1% pentobarbital sodium (60 mg/kg) as previously described (Pang et al., 2020). For the 2-vessel occlusion procedure, bilateral common carotid arteries were gently isolated and ligated with 5–0 silk sutures. The sham group treated the rats with a similar surgery without carotid ligation. Four weeks later, bilateral maxillary molars were extracted from 2-VO rats after being anesthetized with pentobarbital sodium. In the molar loss sham group, little gingival and bone were removed with rongeur from the bilateral maxillary alveolar ridge. Surgical photos were shown in the Supplementary material (Supplementary Figure 1). The general condition and body weight of each rat were monitored for the next 8 weeks.

### Morris water maze test

Morris water maze test (MWM) was performed 7 weeks after molars extraction to analyze the cognitive function according to the previously described (Pang et al., 2020). A circular tank (diameter 150 cm and depth 60 cm) covered with black tape was filled with 20 ± 1^°^C water. The escape platform (diameter of 10 cm and 28 cm in height) was fixed at 1 cm beneath the water surface in the middle of the first quadrant. The indoor environment and the observer remained fixed throughout the experiment.

The Positioning navigation training was performed 7 weeks after molar extraction for four consecutive days. Rats were trained to locate the hidden platform in two trials per day. The escape latency was recorded as 60 s in such cases. When the rats reached the platform within 60 s, they remained on the platform for 10 s. If the rats failed to find the platform within 60 s, they were gently guided to the platform and left on it for 10 s. On the fifth day, the platform was removed for probe trial. The escape latency, time of first passing the platform (TFPP), the frequency of passing the platform (FPP), and the percentage of time spent in the target quadrant (tP/tT) were recorded for each rat within 60 s.

### Nissl staining

Animals were anesthetized with chloral hydrate and perfused with saline followed by 4% paraformaldehyde. The separated brains were embedded in paraffin. After dewaxing and hydration procedures, the 5 μm hippocampus slices were immersed in Nissl staining solution (Methylene Blue) for 10 min. Then, the sections were rinsed with distilled water, dehydrated in ethanol, and cleared in xylene. Images in the CA1 and CA3 regions of the hippocampus were captured with a light microscope (Olympus BX61, Japan).

### Terminal deoxynucleotidyl transferase dUTP nick end labeling assay

Neuronal apoptosis was analyzed by terminal deoxynucleotidyl transferase dUTP nick end labeling (TUNEL) assay with an *in situ* cell death detection kit following the instructions (One Step TUNEL Apoptosis Assay Kit, Beyotime China). The 5 μm brain sections were deparaffinized and incubated with the 10 mg/ml proteinase K (Biomed, China) for 10 min at 37°C. After washing with PBS, the sections were incubated with the TUNEL reaction mixture at 37°C for 1 h. Then, the sections were sealed with a quench-proof tablet containing DAPI. The positive TUNEL cells were visualized on the fluorescence microscope (Olympus BX61, Japan). Five random fields of each image were examined at an objective magnification of ×200, and the positive rate of TUNEL was quantified *via* ImageJ.

### Quantitative real time-PCR analysis

Total RNA was extracted from the hippocampus by TRIZOL (Thermo Fisher Scientific, USA). cDNA was synthesized by reverse transcribing 1 μg RNA with a cDNA Reverse Transcription Kit (CoWin Biosciences, China). Quantitative real time-PCR (qRT-PCR) was conducted using SYBR Green PCR Master Mix (CoWin Biosciences, China) on a Step One Plus Real-Time PCR System (Biorad, USA). All primers, listed in Table 1, were designed and compounded by Sangon Biotech (Shanghai, China).

**TABLE 1:** The primers used in qRT-PCR.

Gene name	Primer	Sequence
p38MAPK	Forward	AGTCCTATCCACGCACCTCA
	Reverse	GTCCCGTTTCCTGCACCAC
NFκB	Forward	ACGATCTGTTTCCCCTCATC
	Reverse	TGCTTCTCTCCCCAGGAATA
caspase3	Forward	ACTGGAAAGCCGAAACTCTTCATCA
	Reverse	GGAAGTCGGCCTCCACTGGTATC
iNOS	Forward	CAGCATCCACGCCAAGAACG
	Reverse	CACAGTTTGGTCTGGCGAAG
GAPDH	Forward	TTCCTACCCCCAATGTATCCG
	Reverse	CCACCCTGTTGCTGTAGCCATA

### Immunohistochemistry and histology

immunohistochemistry and histology (IHC) staining was performed to detect the expression of p38MAPK, NFκB, caspase 3, and GFAP. After the antigens were repaired, all sections were incubated with the primary antibody for p38MAPK (1:300, Bioss), NFκB (1:300, Abcam), caspase 3 (1:200, Abcam), and GFAP (1:1000, Proteintech) overnight at 4°C after blockage of endogenous peroxidases. Next, the sections were incubated with the secondary antibody (Maixin, China) at 37°C for 30 min. Finally, slides were evaluated using the brown DAB precipitate (Maixin, China) and were performed with hematoxylin. All sections were observed under the microscope (Olympus BX61, Japan) to randomly select three regions for MOD analysis.

### Immunofluorescence analysis (IF)

After the antigens were repaired, the sections were incubated with GFAP (1:200, Proteintech) and p38MAPK (1:200, Bioss) overnight at 4°C. Balance at room temperature for 30 min, the fluorescent secondary antibody (1:500) was incubated at room temperature for 2 h, and nuclei were counterstained with DAPI. Images were taken by microscope (Olympus BX61, Japan).

### Intrathecal injection of drugs

p38MAPK inhibitor (SB203580) was used to verify the role of the p38MAPK signal pathway. The 2-VO MO rats were divided into two groups: the 2-VO MO (vehicle control, *n* = 6) group and 2-VO MO SB203580 (p38MAPK inhibitor, *n* = 6) group. Rats in the SB203580 group received intrathecal injections of a 10-μl solution containing 10 μg of the p38MAPK inhibitor, and vehicle-injected rats received an equal volume of DMSO. One week after intrathecal injection, animals were examined in the MWM and brain tissues were taken for TUNEL and IHC staining.

### Statistical analysis

All statistical analysis was performed using SPSS 22.0 software. The graphs were prepared by GraphPad Prism (version 6.0, GraphPad Software). Multiple comparisons were analyzed by one-way ANOVA test. All data were presented as mean ± standard deviation (SD). *p* < 0.05 was considered as statistically significant.

## Results

### Molar loss exacerbated cognitive impairment in 2-VO rats

**FIGURE 2:**
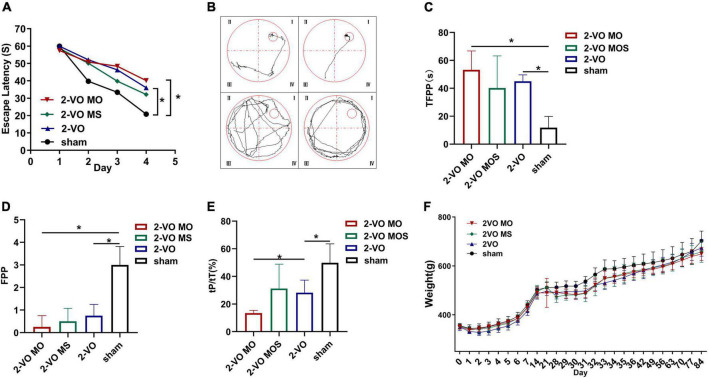
Evaluation of memory and learning function *via* Morris water maze test. **(A)** Changes of escape latency in learning trial. **(B)** Representative traces of the probe test in the water maze test; **(C)** the time of first passing the platform (TFPP). **(D)** The frequency of passing the platform (FPP) in each group. **(E)** Time spent in the target quadrant of four groups in the probe trial. **(F)** Body weight changes in groups (All values are expressed as mean ± SD, *n* = 10, *p < 0.05).

After 8 weeks of modeling, the Morris water maze was used to evaluate the spatial learning and memory ability of the rats in each group. As shown in Figure 2A, the escape latency of rats in each group was gradually shortened with increased learning times. On the third and fourth days, the average escape latency of rats in the 2-VO group and 2-VO MO group was significantly longer than in the sham group (*p* < 0.05). On the fourth day, the escape latency of the 2-VO MO group was longer than that of the 2-VO MS group, but there was no statistical difference (*p* > 0.05).

Representative examples of typical swimming paths indicate that molar loss decreased the frequency of platform crossing and searched for the platform mainly in random form or edge form (Figure 2B). Morris water maze spatial exploration results showed that TFPP values in the 2-VO MO group and 2-VO group were higher than that in the sham group, while FPP values were lower (*p* < 0.05). Compared with the 2-VO MS group, the TFPP value of the 2-VO MO group increased, and the FPP value decreased with no statistical significance (*p* > 0.05, Figures 2C,D). In Figure 2E, the tP/tT ratio in the 2-VO group was significantly lower than in the sham group (*p* < 0.05). 2-VO MO rats spent significantly less time in the platform region than 2-VO MS and sham rats (*p* < 0.05). As shown in Figure 2F, the body weight of rats decreased to varying degrees after the two-vessel occlusion surgery or molars extraction. From 5 days after the surgery and extraction, the body weight of all groups recovered gradually. And the weight of all the groups increased gradually in a similar trend. There was no significant difference between the body weight in each group (*p* > 0.05).

### Molar loss exacerbated 2-VO induced neuronal damage in hippocampus

**FIGURE 3:**
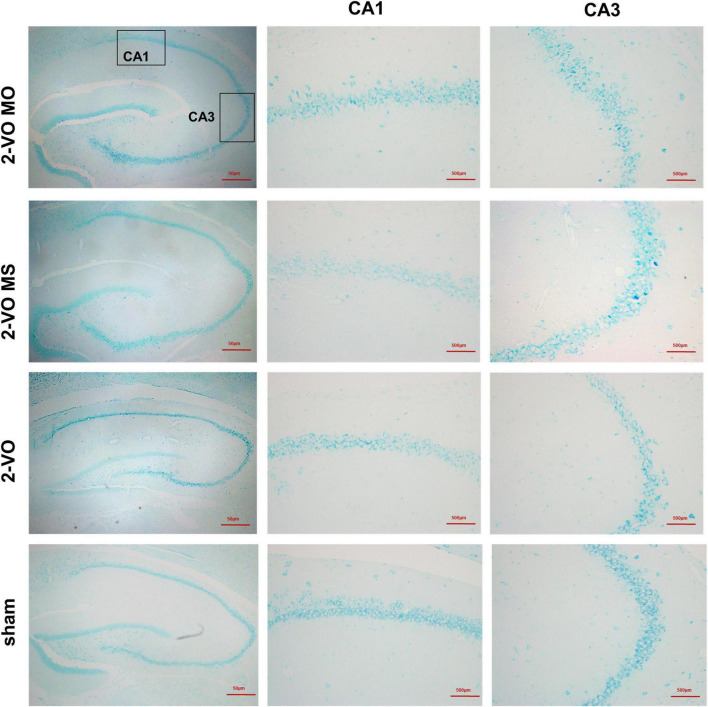
Effects of molar loss on the CA1 and CA3 neurons morphology in 2-VO rats’ hippocampus (Nissl’s staining, ×200, *n* = 4).

Chronic cerebral ischemia can act on neurons to result in pathologic changes, which lead to cognitive impairment. The hippocampal neuron structure was examined in each group to determine whether molar loss exacerbated 2-VO-induced neuron damage. Nissl staining results showed that the CA1 and CA3 region neurons exhibited a regular arrangement, distinct edges, and clear nucleus and nucleolus in hippocampal cells in the sham group (Figure 3). While in the 2-VO MO group, the hippocampal cells were shown to be disorganized, structure ambiguous, nucleus condensate, and deeply staining. These changes were observed more easily in 2-VO MS and 2-VO groups.

### Increased numbers of apoptotic cells in the hippocampus of 2-VO rats following molar loss

**FIGURE 4:**
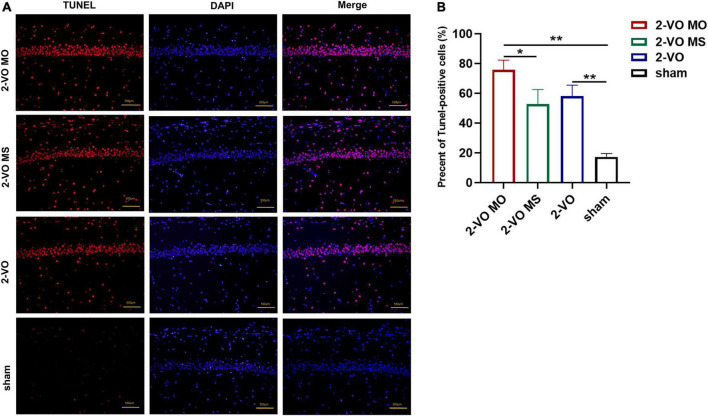
TUNEL staining of CA1 region of rat hippocampus. **(A)** Cell nuclei (blue), the red spots represent TUNEL-positive cells. **(B)** The TUNEL-positive rate in CA1 quantification (×200, the positive rate is expressed as mean ± SD, *n* = 4, *p < 0.05, **p < 0.01).

In this study, the neuronal apoptosis in the hippocampus was verified with TUNEL labeling. There were a few TUNEL-positive cells in the sham group in the hippocampus. Quantitative analysis showed that the number of apoptotic cells in the 2-VO MO group and 2-VO group was significantly higher than in the sham group (Figure 4, *p* < 0.01). Specifically, 2-VO MO rats exhibited substantially higher levels of apoptosis than 2-VO MS rats (Figure 4, *p* < 0.05).

### Molar loss further activated the p38MAPK/NFκB mRNA expression in 2-VO rats

**FIGURE 5:**
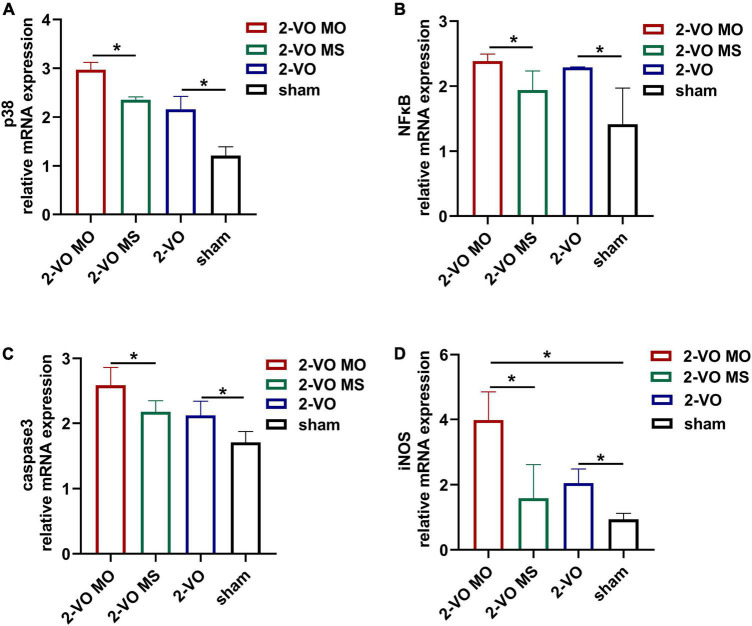
Effects of molar loss on the mRNA expression of p38MAPK **(A)**, NFκB **(B)**, caspase3 **(C)**, and iNOS **(D)** in the rat hippocampus. All values are expressed as mean ± SD, *p < 0.05, *n* = 6).

To further explore the mechanisms of the molar loss-associated in the hippocampus apoptotic response, the p38MAPK, NFκB, caspase 3, and iNOS mRNA expressions were detected and presented in Figure 5. Compared with the sham group, chronic cerebral ischemia increased the expression of p38MAPK, NFκB, caspase 3, and iNOS mRNA in the hippocampus (*p* < 0.05). In addition, molar loss further induced upregulation of these genes in the hippocampus of 2-VO rats (*p* < 0.05).

### Molar loss induced further upregulation of apoptosis-related proteins in 2-VO rats

**FIGURE 6:**
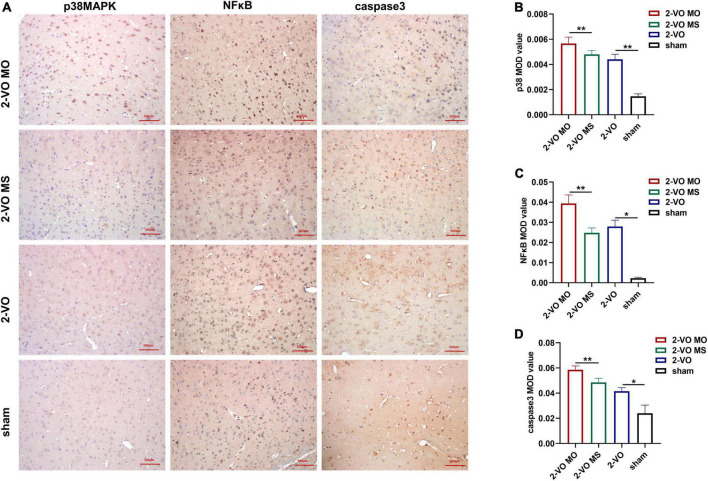
Effects of molar loss on the protein expression of p38MAPK, NFκB and caspase3 in the CA1 region of rat hippocampus. **(A)** Representative IHC images of p38MAPK, NFκB, and caspase 3 (IHC, ×200). **(B–D)** The MOD values are expressed as mean ± SD, *n* = 4, *p < 0.05, **p < 0.01.

Immunohistochemistry and histology verified the activation of apoptosis-related proteins. As shown in Figure 6, compared with the sham group, the expressions of p38MAPK, NFκB, and caspase 3 were significantly increased in the hippocampus of 2-VO rats (*p* < 0.01). In addition, higher expression levels of p38MAPK, NFκB, and caspase 3 were observed in the 2-VO MO rats’ hippocampus than those in the 2-VO MS rats (*p* < 0.01).

### Molar loss induced further upregulation of GFAP expression in 2-VO rats

**FIGURE 7:**
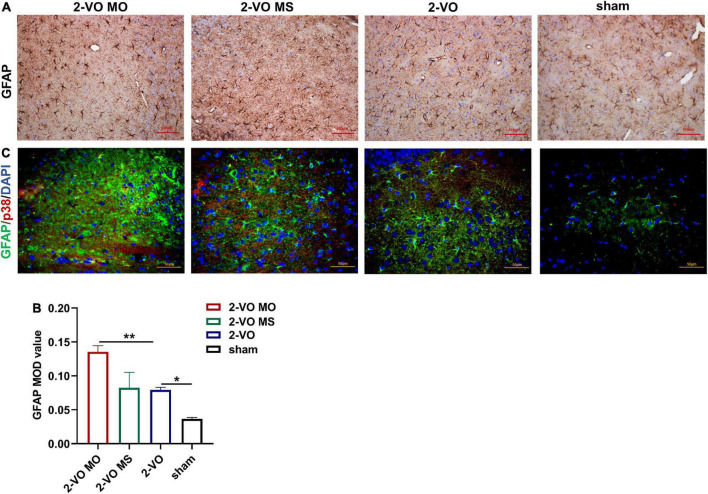
Effects of molar loss on GFAP in the rat hippocampus. **(A,B)** IHC staining showed the expression of GFAP (IHC, ×200, *n* = 4, MOD values are expressed as mean ± SD, *p < 0.05, **p < 0.01). **(C)** Double immunofluorescent staining revealed colocalization of GFAP (green) and p38MAPK (red) proteins in the rat hippocampus, and the nuclei were in blue (magnification ×400).

GFAP is a marker of astrocyte activation. The expression of GFAP was determined with DAB. In Figures 7A,B, the expression of GFAP in the hippocampus of the 2-VO group was significantly higher than sham group (*p* < 0.05). And the higher expression of GFAP was observed in the 2-VO MO rats’ hippocampus than in the 2-VO rats (*p* < 0.01). The reactive astrocytes in 2-VO MO rats’ hippocampus were larger, with thicker, and more clearly visible processes compared with other groups. Then, we carried out the colocalization of p38MAPK and GFAP following. The p38MAPK-positive cells were labeled with the astrocyte marker GFAP in the hippocampus of 2-VO MO rats (Figure 7C).

### SB203580 reduced cognitive impairment and apoptosis of hippocampal neurons in 2-VO rats following molar loss

**FIGURE 8:**
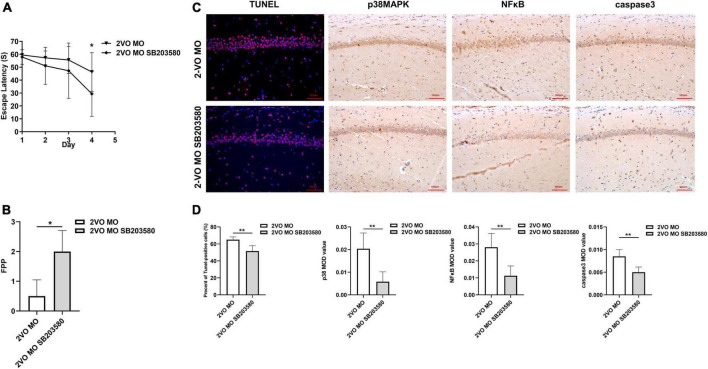
Effects of SB203580 on cognitive impairment and apoptosis of hippocampal neurons in 2-VO rats following molar loss. **(A)** Changes in escape latency in learning trial (*p < 0.05). **(B)** The frequency of passing the platform (FPP) in each group (*p < 0.05). **(C,D)** TUNEL staining of CA1 region of rat hippocampus cell nuclei (blue), the red spots represent TUNEL-positive cells (×200, the positive rate is expressed as mean ± SD, *n* = 6, **p < 0.01). IHC staining showed the expression of p38MAPK, caspase3, and NFκB (IHC, × 200, *n* = 6, MOD values are expressed as mean ± SD, **p < 0.01).

One week after intrathecal injection, the Morris water maze was used to evaluate the spatial learning and memory ability of the rats in each group. As shown in Figure 8A, the escape latency of rats in each group was gradually shortened with increased learning times. On the fourth day, the average escape latency of rats in the 2-VO MO SB203580 group was significantly shorter than in the 2-VO MO group (Figure 8A, *p* < 0.05). Morris water maze spatial exploration results showed that FPP values in the 2-VO MO SB203580 group were higher than that in the 2-VO MO group (Figure 8B, *p* < 0.05). TUNEL-positive cells were significantly decreased in the 2-VO MO SB203580 group (Figures 8C,D, *p* < 0.05). Compared with 2-VO MO rats, the expression of p38MAPK, NFκB, and caspase 3 was significantly induced by SB203580 (Figures 8C,D, *p* < 0.05). It suggests that p38MAPK pathway activation is involved in molar loss exacerbated 2-VO induced cognitive impairment.

## Discussion

In recent decades, human teeth, tools used for chewing, have also been considered essential for the nutrition and health of the human. Teeth loss and occlusion dysfunction affect brain function and promote the progress of cognitive decline (Nakamura et al., 2021). The latest systematic meta-analysis found that tooth loss was one of the independent risk factors for cognitive dysfunction, and the risk of cognitive impairment was positively correlated with the number of tooth loss (Cerutti-Kopplin et al., 2016; Fang et al., 2018; Wang et al., 2022). In addition, studies have shown that timely denture repair of missing teeth can slow down the development of cognitive decline related to tooth loss (Lopez-Chaichio et al., 2021). VaD is a syndrome of severe cognitive dysfunction caused by ischemic stroke, hemorrhagic stroke, and other cerebrovascular diseases (Kalaria et al., 2016). It has been found that clenching the jaw muscles can increase cardiac output and stimulate local blood flow into the brain (Hasegawa et al., 2007; Zhang et al., 2012). Our previous study found that the loss of molars caused cognitive impairment, a decrease in CBF, and neuronal apoptosis in rats (Luo et al., 2019). Based on this, we established a molar loss model in 2-VO rats to explore the effect of molar loss on VaD and the possible mechanism. The ligation of the common bilateral arteries could cause global ischemia of the brain. Bilateral maxillary molars were removed to eliminate occlusal contacts, and all the tooth structure visible above the gum was removed if the root was fractured (Pang et al., 2020). In Figure 1F, the body weight of rats in all groups increased gradually in a similar trend. There was no significant difference in the body weight in each group. These data were consistent with the literature and our previous studies. In this study, we removed bilateral maxillary molars to simulate loss of occlusal support without causing malnutrition in rats. Therefore, we can exclude the nutritional deficiency factor after molar loss in this study.

Morris water maze test was then performed for the evaluation of learning and memory function. All the results indicated the aggravation of molar loss on the damaged learning and memory abilities in 2-VO rats. In this study, the learning ability of rats was evaluated by a positioning navigation experiment. The decreased trend and amplitude of escape latency in the 2-VO MO and 2-VO groups were significantly shorter than those in the sham group, indicating that the learning ability of rats decreased when chronic cerebral ischemia occurred. The latency of rats in the 2-VO MO group was longer than that in the 2-VO group, but the difference between 2-VO MO and 2-VO rats was insignificant. We speculate that a compensatory mechanism was established in the long-term chronic cerebral ischemia process to help improve cerebral ischemia injury. In addition, the latency was the length of time for rats to escape to the platform, which was also closely related to the swimming speed of rats.

The swimming search strategy, TFPP, FPP, and tP/tT in the spatial exploration experiment can reflect the memory ability of rats. The results showed that the rats in a sham group could find and cross the platform area repeatedly in a short time, and their movement strategies were mostly tendentious or linear. The rats in 2-VO MO and 2-VO groups could not accurately memorize the position of the platform, and it took a long time to cross the original platform area, and the number of crossings was reduced. However, there were more movements in other quadrants, and the trajectory was mainly random or marginal, indicating that they did not form good spatial memory. The tP/tT of rats in the 2-VO MO group was lower than that in the 2-VO group, with significant statistical differences, indicating that molar loss further aggravated the spatial memory ability of rats with chronic cerebral ischemia. However, there was no significant difference in TFPP and FPP between the 2-VO MO group and the 2-VO group. It may be that the rats in 2-VO MO and 2-VO groups have been compensated to some extent after 12 weeks of chronic cerebral ischemia. With the establishment of collateral circulation and posterior circulation, this injury may be compensated to extent. These results suggest that the loss of molars may lead to further aggravation of cognitive dysfunction in rats with chronic cerebral ischemia, which needs further study. Tooth loss is associated with impairment of hippocampus-dependent cognition (Katano et al., 2020). The pathological changes of the hippocampal alterations, however, remains unclear.

With the bio informative analyses, chronic cerebral ischemia-induced hippocampal injury is closely related to apoptosis and involves multiple signaling pathways such as the MAPK pathway (Supplementary Figure 2). The pathogenesis of VaD is related to the p38MAPK signaling pathway that induces neuronal apoptosis and oxidative stress (Lu et al., 2019). After stress-induced phosphor activation, p38MAPK activates gene expression patterns that can alternately induce proliferation, differentiation, cytokine synthesis, or caspase 3-dependent apoptosis. Caspase 3, one of the most critical proteases in the caspase family, can directly induce apoptosis (Velier et al., 1999). Caspase 3 is a marker of neuronal apoptosis, and the expression of caspase 3 inhibition reduces neural apoptosis (Zhang et al., 2021). Bcl-2 is an upstream inhibitor of caspase 3, and caspase 3 is the primary effector of apoptosis. In this experiment, through Nissl staining and TUNEL staining, it was found that neurons in hippocampal CA1 and CA3 areas of 2-VO MO rats were disordered, and apoptotic cells were significantly increased compared with those in 2-VO or 2-VO MS rats. In this study, the results of IHC and PCR showed the expression of p38MAPK and caspase 3 in the 2-VO group was higher than that in the sham group, and the expression of p38MAPK and caspase 3 in the 2-VO MO group was higher than that in 2-VO MS group. With chronic cerebral ischemia, glial cells changes in morphology, number, and molecular biology. Astrocytes and microglia are involved in the inflammatory response after chronic cerebral ischemia, and nitric oxide (NO) and reactive oxygen species (ROS) are involved in brain injury (Zhou Y. et al., 2020). The expression of GFAP in the hippocampus of the 2-VO group was significantly higher than in the sham group. And the higher expression of GFAP was observed in the hippocampus of 2-VO MO rats than those in the 2-VO rats. The active astrocytes in 2-VO MO rats’ hippocampus are larger, with thicker, and more clearly visible processes compared with other groups. Our double-labeling result demonstrated that activation of p38MAPK occurred in astrocyte cells of molar loss in 2-VO rats. The expression of p38MAPK in glial cells has also been reported in other orofacial pain models and the progression of tissue damage resulting from cerebral ischemia models (Cao et al., 2013). As a preliminary step, the phenotypic responses and descriptive analyses of molecular were explored and discussed in this study. It suggests that cell apoptosis is involved in learning and memory impairment development in rats with persistent chronic cerebral ischemia by activating p38MAPK and caspase 3.

It has been reported that the p38MAPK inhibitors quercetin and SB202190 can block the p38MAPK pathway to inhibit caspase 3 activation, protect hippocampal neurons from chronic ischemic injury, and rescue spatial learning and memory deficits (Chen et al., 2009; Yang et al., 2013). To further verify whether the p38MAPK pathway has regulatory effects in 2-VO rats, a p38MAPK inhibitor (SB203580) was intrathecally injected *in vivo* study. The results showed that SB203580 reduced cognitive impairment and apoptosis of hippocampal neurons in 2-VO rats following molar loss. In addition, SB203580 significantly repressed p38MAPK, NFκB, and caspase 3 expression in hippocampus. It suggests that p38MAPK pathway activation is involved in molar loss exacerbated 2-VO-induced cognitive impairment. NFκB activation in neurons promotes their survival and plasticity, whereas NFκB activation in glial cells enhances neuronal death (Chang and Huang, 2006; Lee et al., 2017). After cerebral ischemia injury, NFκB, activated by phosphorylated inhibitory proteins, enters the nucleus to promote the expression of iNOS and tumor necrosis factor-α, causing damage to cerebral ischemia tissue by the vicious circle (Liang et al., 2019). The qRT-PCR and IHC showed that the expression of NFκB in the 2-VO group was higher than that in the sham group (*p* < 0.05), and the expression of NFκB in the 2-VO MO group was significantly higher than that in the 2-VO MS group. It indicated that the activation of NFκB is enhanced in chronic cerebral ischemia, which may further promote the release of inflammatory factors and cause cerebral ischemia damage. Molar loss may further promote the apoptosis of hippocampal neurons and aggravate cognitive impairment by upregulating the expression of NFκB. NO is closely associated with ischemia/hypoxia-induced neuronal injury (Chen et al., 2009) and is related to the learning and memory capacities of VaD rats (Fernandez et al., 2012). The activated glial cells promote the expression of iNOS and the increase of NO, which can affect mitochondrial function by increasing excitotoxicity, leading to cell necrosis or apoptosis, and mediating inflammatory cascade reaction through the NFκB pathway (Van Hemelrijck et al., 2005). Our previous studies have found that the loss of molars leads to a decrease in CBF in the hippocampus of rats and increases the expression of NO and iNOS in the hippocampus (Pang et al., 2015, 2020; Luo et al., 2019). In the present study, qRT-PCR confirmed that molar loss led to further upregulation of iNOS mRNA expression. We also found that the trend of iNOS was consistent with p38MAPK. These results suggest that loss of occlusal support may further enhance apoptosis by inducing iNOS and NFκB expression.

There are several potential underlying mechanisms regarding the relationship between the molar loss and cognitive impairments, such as diminished sensory input, decreased CBF, impaired cholinergic neurotransmission, or increased stress responses. This study found that the spatial learning and memory of Wistar rats were impaired after molar loss with 2-vessel occlusion. The apoptosis of hippocampal interneurons could have many implications on hippocampal-dependent behaviors (Bird et al., 2018). Interestingly, we found that apoptosis and related molecular parameters were significantly altered in the hippocampus of rats with chronic cerebral ischemia and molar loss, while only the tP/tT changes were statistically significant in behavioral tests in this study. We speculate that the possible reason may be due to the existence of a certain compensatory mechanism in rats, which needs further investigation. The CA1 area is the functional area most closely related to learning and memory in the hippocampus, and it is very sensitive to ischemia and hypoxia. Various studies have found the apoptosis of neurons in the hippocampus CA1 region of VaD rats, which is the possible pathological basis for the occurrence of VaD. Our previous study found that the loss of molars caused cognitive impairment, a decrease in CBF, and neuronal apoptosis in rats. Molar loss may induce pathological changes in the hippocampus early by reducing CBF. However, with the establishment of collateral circulation and posterior circulation, this injury may be compensated to an extent. This may explain the inconsistent behavioral phenotypes with pathological changes.

## Conclusion

In summary, molar loss aggravates the damage to spatial learning and memory ability caused by chronic cerebral ischemia in rats. The mechanism may be related to reduced CBF in the hippocampus and further activation of the p38MAPK-NFκB-caspase 3 pathway to induce neuronal apoptosis (Graphical abstract created by BioRender^1^). This study may provide a mechanism partially explaining deficits in hippocampal and cognitive in 2-VO rats with molar loss.

## Data availability statement

The original contributions presented in this study are included in the article/Supplementary material, further inquiries can be directed to the corresponding authors.

## Ethics statement

The animal study was reviewed and approved by the Animal Ethical and Welfare Committee of Capital Medical University School of Stomatology.

## Author contributions

YL, XT, and QJ: conceptualization. YL: data curation, formal analysis, and writing – original draft. QJ: funding acquisition and project administration. YL, QP, QW, and BL: methodology. XT and QJ: supervision and writing – review and editing. All authors have read and agreed to the published version of the manuscript.

## Footnotes

1
https://biorender.com
