# Corrigendum: Molar loss further exacerbates 2-VO-induced cognitive impairment associated with the activation of p38MAPK/NFκB pathway

**DOI:** 10.3389/fnagi.2022.1099785

**Published:** 2022-11-28

**Authors:** Yunping Lu, Qian Pang, Qianqian Wu, Bin Luo, Xiaofei Tang, Qingsong Jiang

**Affiliations:** ^1^Department of Prosthodontics, Beijing Stomatological Hospital, School of Stomatology, Capital Medical University, Beijing, China; ^2^Department of Stomatology, People's Hospital of Beijing Daxing District, Capital Medical University, Beijing, China; ^3^Division of Oral Pathology, Beijing Institute of Dental Research, Beijing Stomatological Hospital, School of Stomatology, Capital Medical University, Beijing, China

**Keywords:** molar loss, vascular dementia, cognitive impairment, apoptosis, p38MAPK

In the published article, there was an error in the Funding statement. The funding number for Beijing Stomatological Hospital, Capital Medical University Young Scientist Program was incorrect. The corrected Funding statement appears below.

“This study was supported by the National Natural Science Foundation of China (Nos. 81771094 and 82170980) and the Beijing Stomatological Hospital, Capital Medical University Young Scientist Program (No. YSP202106).”

The authors apologize for this error and state that this does not change the scientific conclusions of the article in any way. The original article has been updated.

## Publisher's note

All claims expressed in this article are solely those of the authors and do not necessarily represent those of their affiliated organizations, or those of the publisher, the editors and the reviewers. Any product that may be evaluated in this article, or claim that may be made by its manufacturer, is not guaranteed or endorsed by the publisher.

