# Supplementary material for: Molar loss further exacerbates 2-VO-induced cognitive impairment associated with the activation of p38MAPK/NFκB pathway
Source: Front Aging Neurosci. 2022 Nov 3;14:930016. doi: 10.3389/fnagi.2022.930016 (PMC9669382; doi:10.3389/fnagi.2022.930016)
Supplement: Supplementary file 1 [file Data_Sheet_1.docx]

Supplementary Material

# Supplementary Figures


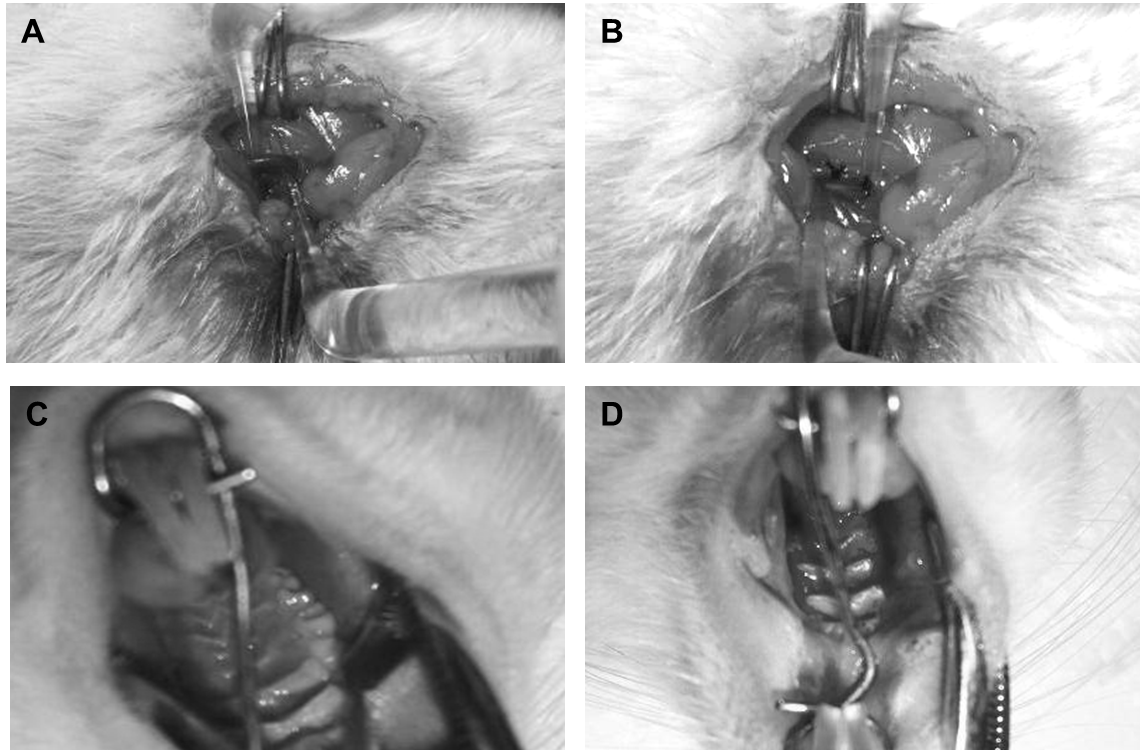


**Figure 1.** **The establishment of chronic cerebral ischemia and occlusal support loss in rats.** (A, B) Bilateral common carotid arteries were separated and ligated. (C.D) Extraction of all molars in bilateral maxillary.


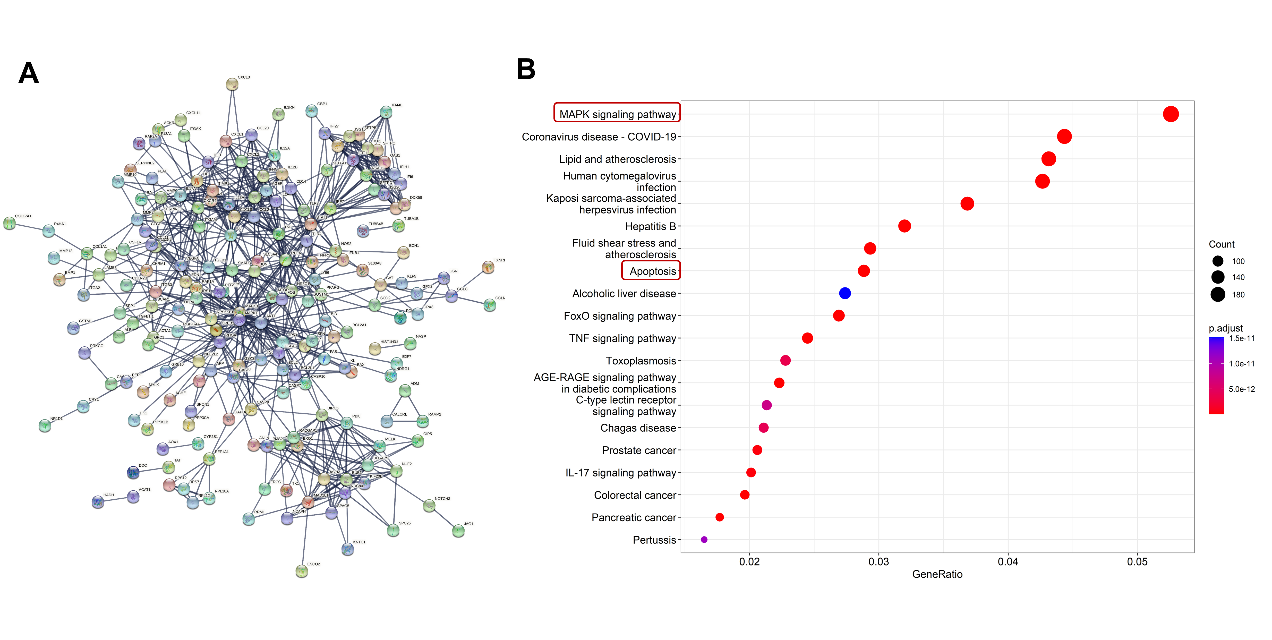


**Figure 2. With the bio informative analyses chronic cerebral ischemia-induced hippocampal injury is closely related to apoptosis and involves multiple signaling pathways such as MAPK pathway. Data from the Comparative Toxicogenomics Database (CDT).** (a) Subnetwork of VaD related genes from the CTD database and PPI network; (b) Pathway enrichment analysis of candidate genes VaD: The y‑axis represents KEGG‑enriched terms. The x‑axis represents the fold of enrichment. The size of the dot represents the number of genes under a specific term. The color of the dots represents the adjusted P-value.


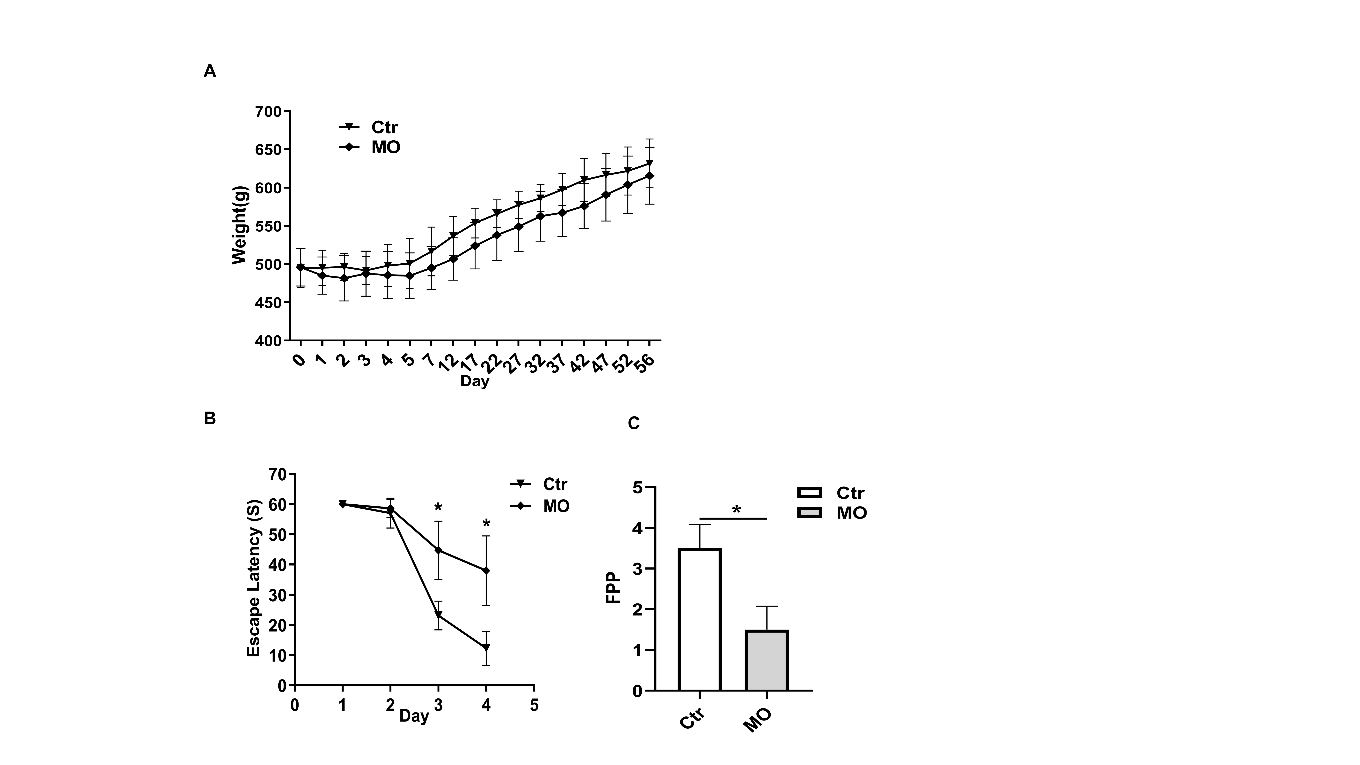


**Figure 3.** **The results of Morris water maze of occlusal support loss in rats. (A)** Body weight changes in groups. **(B)** Changes of escape latency in learning trial. **(C)** the frequency of passing the platform (FPP) in each group. (All values are expressed as mean ± SD, n = 10, *p < 0.05.)


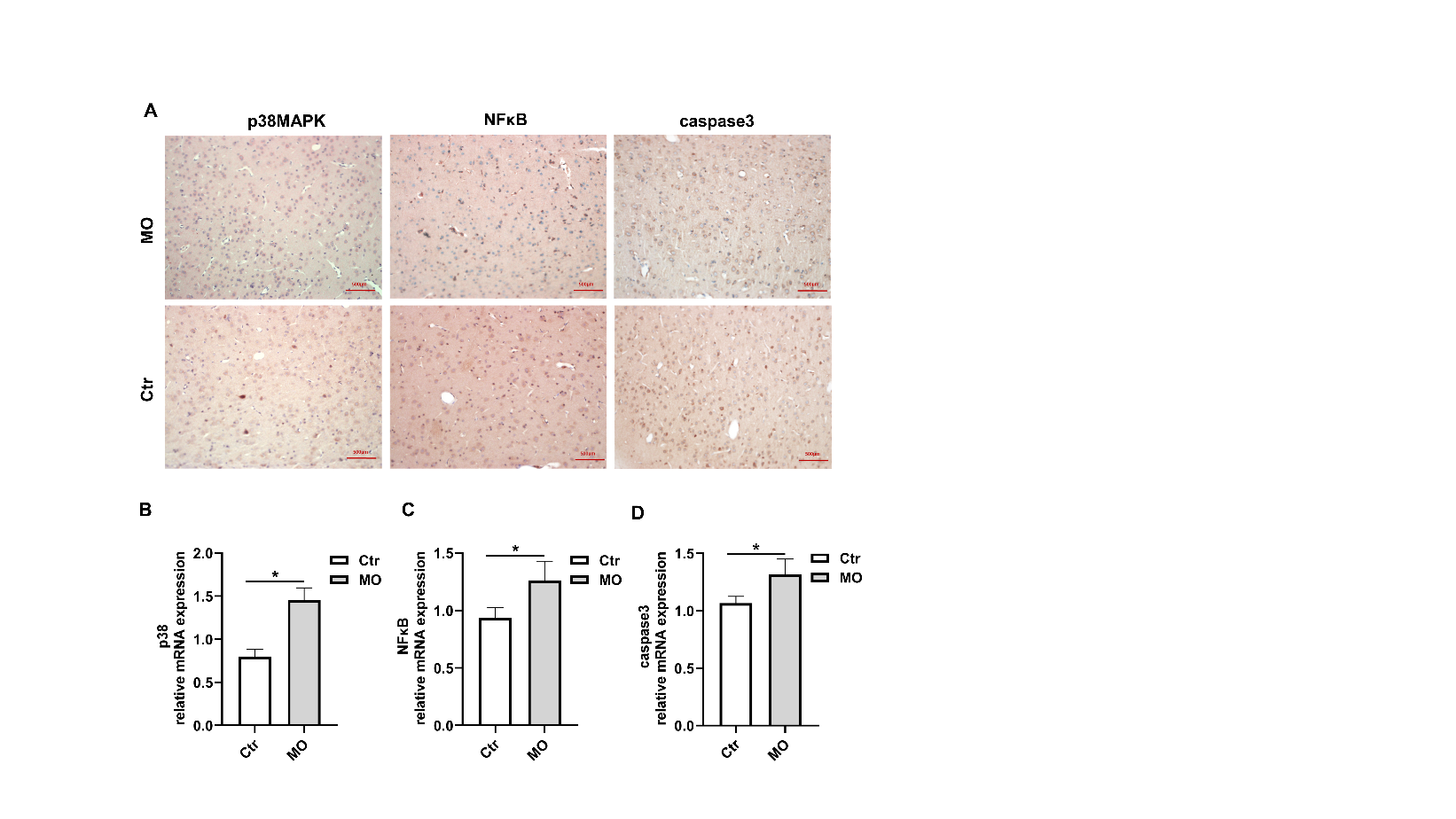


**Figure 4. (A)** Effects of molar loss on the protein expression of p38MAPK, NFκB, and caspase 3 in the CA1 region of rat hippocampus. (IHC, ×200). **(B)** Effects of molar loss on the expression of p38MAPK, NFκB, and caspase 3 in the rat hippocampus. mRNA (All values are expressed as mean ± SD, *p<0.05, **p<0.01, n = 6).


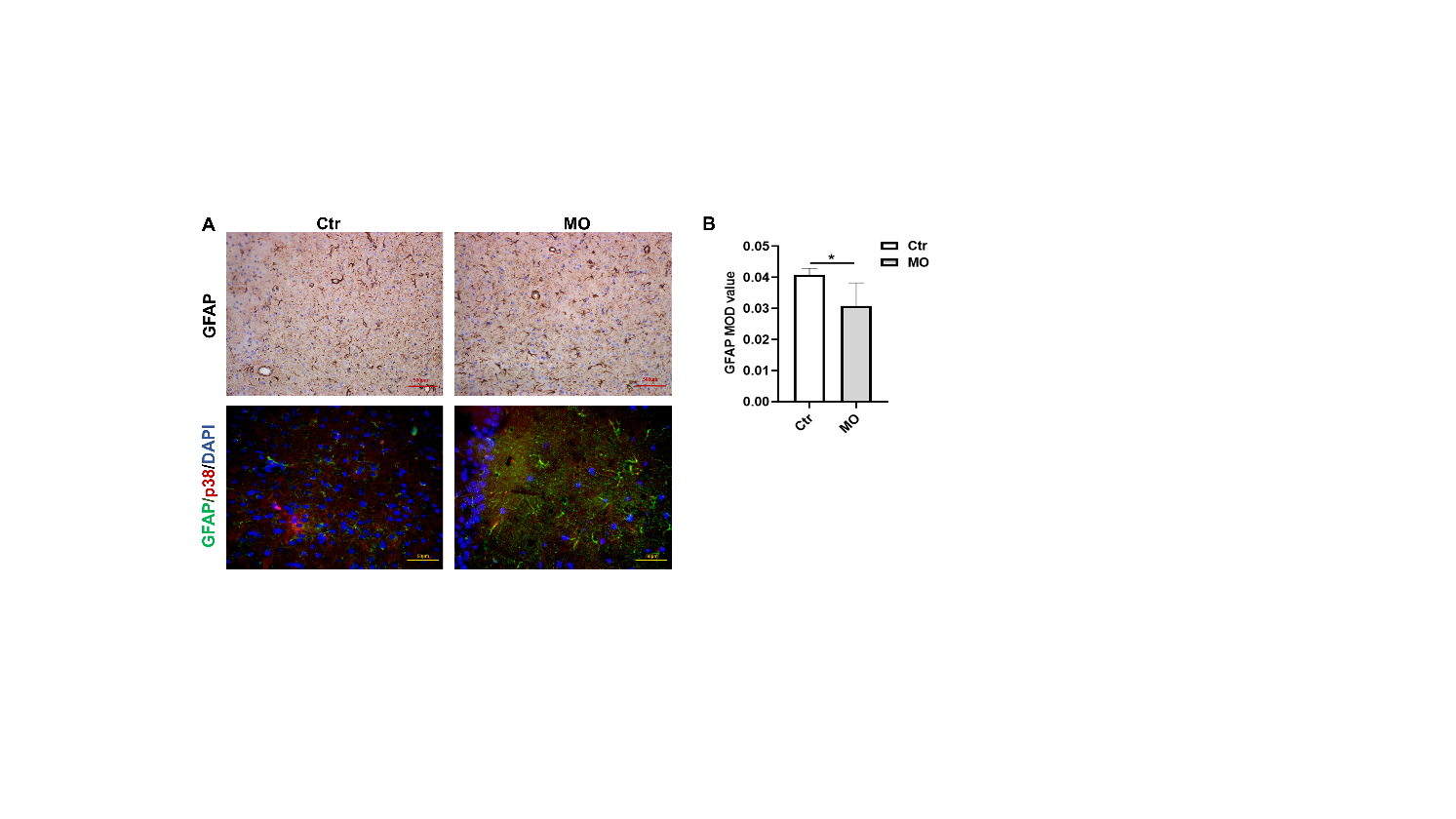


**Figure 5. Effects of molar loss on GFAP in the rat hippocampus.** **(A)** IHC staining showed the expression of GFAP (IHC, ×200); Double immunofluorescent staining revealed co-localization of GFAP (green) and p38MAPK (red) proteins in the rat hippocampus and the nuclei were in blue (magnification ×400). **(B)** MOD values are expressed as mean ± SD, *p<0.05.
